# The Detection of Burn-Through Weld Defects Using Noncontact Ultrasonics

**DOI:** 10.3390/ma11010128

**Published:** 2018-01-13

**Authors:** Zeynab Abbasi, Donald Yuhas, Lu Zhang, Alexandra-Del-Carmen Basantes, Niloofar Nabili Tehrani, Didem Ozevin, Ernesto Indacochea

**Affiliations:** 1 Civil & Materials Engineering Department, University of Illinois at Chicago, Chicago, IL 60607, USA; zabbas5@uic.edu (Z.A.); zhang899@uic.edu (L.Z.); abasan2@uic.edu (A.-D.-C.B.); nnabil2@uic.edu (N.N.T.); jeindaco@uic.edu (E.I.); 2 Industrial Measurement Systems Inc., Aurora, IL 60502, USA; dyuhas@imsysinc.com

**Keywords:** Burn-through, weld bead width, noncontact, air-coupled ultrasonics

## Abstract

Nearly all manufactured products in the metal industry involve welding. The detection and correction of defects during welding improve the product reliability and quality, and prevent unexpected failures. Nonintrusive process control is critical for avoiding these defects. This paper investigates the detection of burn-through damage using noncontact, air-coupled ultrasonics, which can be adapted to the immediate and in-situ inspection of welded samples. The burn-through leads to a larger volume of degraded weld zone, providing a resistance path for the wave to travel which results in lower velocity, energy ratio, and amplitude. Wave energy dispersion occurs due to the increase of weld burn-through resulting in higher wave attenuation. Weld sample micrographs are used to validate the ultrasonic results.

## 1. Introduction

Welding is a key manufacturing process in the fabrication of structural parts or components in many industries such as aerospace, automotive, energy, and shipbuilding. Since welding usually occurs late in the manufacturing process, defects (e.g., burn-through, insufficient penetration, porosity) can have significant negative impact, potentially causing scrapped pieces of high relative value. There are several studies in literature correlating welding parameters with weld defects, as well as online monitoring of the welding process. Some of these studies correlated the effects of weld current, voltage, travel speed, heat input, and shielding gas with the weld defects (lack of fusion, burn-through, weld size, lack of strength) using audio (microphone) data by linear correlation [1] and machine learning [2]. Atabaki et al. [3] identified the factors causing porosity in hybrid laser/arc welding in relation to the stand-off distance between the laser and arc, and the heat input. Zhu et al. [4] monitored the electrical parameters during high-frequency induction brazing to identify the ideal parameters for good welding/brazing quality. Zhang et al. [5] pointed out that a single sensor is insufficient to monitor the weld quality. They proposed multisensor information fusion to improve the robustness of the monitoring system. The data includes spectrometer, welding current, and microphone outputs, and the features in the time domain and frequency domain are extracted to monitor the weld quality. Suder and Williams [6] studied the influence of the operational parameters of laser welding on the weld quality. The process variables include laser power, travel speed, and beam diameter to control the weld penetration depth. Several nondestructive evaluation (NDE) methods currently used in weld inspection include visual inspection, dye penetrant, magnetic particle, radiography, and ultrasonic testing. Compared with the other methods, radiography and ultrasonic testing offer an enhanced examination of the welded product, but these methods require a well-trained operator and are typically applied in postmanufacturing [7].

Ultrasonic inspection has high resolution for detecting defects in the weld; however, the challenge with this method is to use a suitable coupling medium to transfer the ultrasonic wave energy into the material. The coupling media commonly used include water, oil, and ultrasonic gel. There are instances, however, where a coupling liquid cannot be used as in the case of in-situ weld inspection where surface temperature and overall contamination risk can be relatively high [8]. The risk can be eliminated by implementing noncontact, air-coupled ultrasonic transducers; however, the main limitations of air-coupled sensing are attenuation in air and acoustic impedance mismatch at the air/steel interface [9]. These limitations have been addressed by recent developments in the design of a newer generation of air-coupled transducers, along with research progress in the field of noncontact ultrasonics [10,11,12,13]. For instance, Chertov et al. [14] developed a real-time ultrasonic monitoring technique for the quality control of spot welds using an ultrasonic transducer embedded in the welding electrode. The method employed various algorithms and was able to determine the quality of the spot weld.

The Lamb-wave-based approach for airborne ultrasonic testing has been implemented in the literature. Most efforts have concentrated on addressing the insufficient energy transfer of air-coupled transducers. Harb and Yuan [15,16] concluded that the antisymmetric mode A_0_ is the most detectable Lamb mode due to dominant out-of-plane displacement at the air/solid interface. They used a hybrid air-coupled/laser inspection system to investigate the interaction of the A_0_ mode for the detection of delamination in composites. Similarly, Ke et al. [17] simulated a noncontact finite element model to demonstrate the detectability of various defects such as impact damage, disband, and through-thickness holes using the A_0_ wave mode. Kažys et al. [18] studied the interaction of Lamb waves on weld defects present in loaded steel plates. There are more complicated situations in which a Lamb wave might interact with the discontinuities and geometry changes, such as thickness variations, causing mode conversions. For instance, Cho [19] investigated the effect of thickness variation on mode conversion in guided wave ultrasonics. Marcial et al. [20] additionally investigated the influence of guided waves in plates containing Gaussian section variation.

The purpose of this study is to apply air-coupled ultrasonics for detecting burn-through damage, which can be adapted to in-situ inspection of the welding process. The outline of this paper is as follows. The Materials and Methods section describes the characteristics of the welded samples and welding parameters, followed by the analytical background required to identify the excitation angle of a pure Lamb wave mode. Then, the experimental procedure required to detect the weld defect is explained. The Results and Discussion section consists of identifying the Ultrasonic Testing (UT) parameters most sensitive to burn-through damage, supported by the micrographs. The last section includes the conclusions and future work of this study.

## 2. Materials and Methods

### 2.1. Sample Preparation and Weld Procedure

The material used for the welded samples was a 0.48 cm thick A36 carbon steel plate with a chemical composition shown in Table 1. The samples were cut to dimensions of 31 × 15 × 0.48 cm.

**Figure 1:**
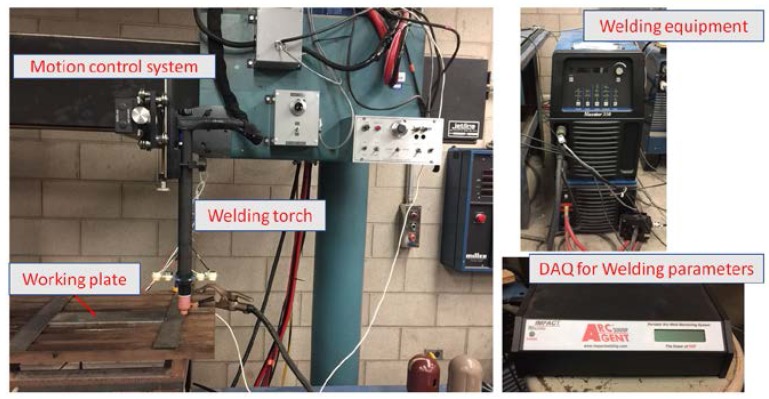
The test setup for the welding process.

Gas tungsten arc welding (GTAW) is used to generate the welded samples. An electric arc struck between the nonconsumable tungsten electrode and a metal workpiece using argon as a shielding gas provides the necessary heat for this welding process. For this welding process, a filler metal may or may not be used. In our studies, no filler metal was used. The DC Miller welding provided by Illinois Tool Works (Illinois Tool Works Inc., Glenview, IL, USA) and a Jetline automated motion system (Miller Electric Manufacturing Co., Appleton, WI, USA) were used to control the travel speed of the welding torch. A Miller Arcagent 3000P system with CenterPoint software provided by ITW was used to collect the real-time welding parameters (i.e., current, voltage, gas flow rate, and power). The entire welding system is shown in Figure 1. 

**Figure 2:**
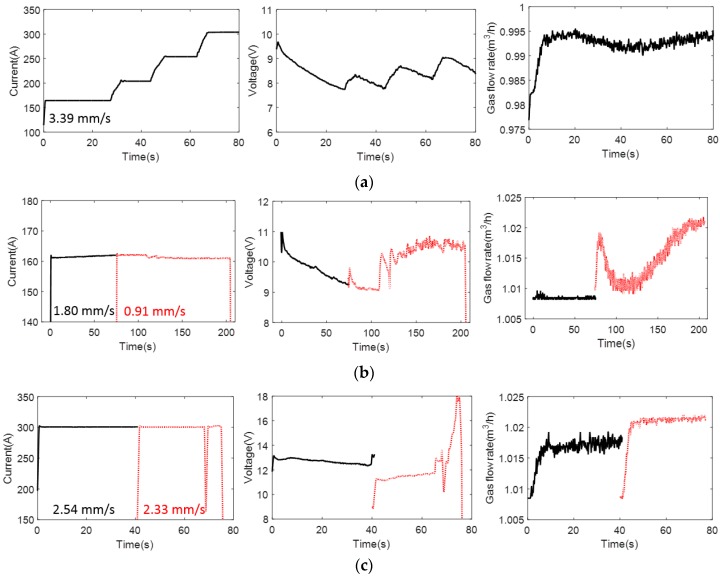
The welding current, voltage, and gas flow for producing a burn-through defect corresponding to (**a**) sample No. 1; (**b**) sample No. 2; and (**c**) sample No. 3.

Burn-through is defined as an undesirable open hole when the base metal completely melts, which can be caused by excessive heat input, improper travel angle, travel speed, and insufficient electrical sickout. In this study, burn-through defects with different excessive penetration levels (reaching up to a complete hole) were introduced by increasing the welding current or reducing the travel speed while keeping the other welding parameters constant. A comparison of the welding process (welding current, voltage, and gas flow rate) between various samples is presented in Figure 2 and Table 2. Weld coupon No. 1 has different weld currents producing four different conditions on the same plate. The variable for weld coupons Nos. 2 and 3 is travel speed—the slower the travel speed is, the higher the heat input is. The difference between the two weld coupons is weld current. Weld coupon No. 3 has a higher current with more severe burn-through damage. Also, the heat inputs presented in Table 2 are calculated according to the Section 4 (Welding, Brazing, and Fusing Qualifications) of ASME Boiler and Pressure Vessel Code using the following Equation (1):

(1) $$Heat\;input\;\left(kJ/mm\right)=\frac{Voltage\left(V\right)\times Amperage\left(A\right)\times 60}{Travel\;speed\left(mm/s\right)\times 1000}\;.$$

### 2.2. Lamb Wave Detection Using Air-Coupled Transducers

**Figure 3:**
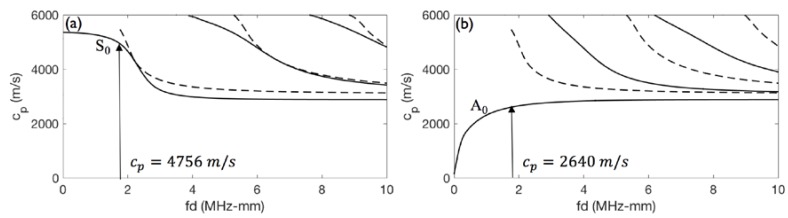
Dispersion curve corresponding to steel plate for (**a**) symmetric modes and (**b**) antisymmetric modes.

The A_0_ mode is the most detectable Lamb mode in airborne ultrasonic testing due to dominant out-of-plane displacement at the air/solid interface. Therefore, the first step in the generation of Lamb waves using air-coupled transducers is to identify the angle required to create a pure Lamb wave mode. Snell’s law suggests that by controlling the angle of the incident wave, different Lamb wave modes can be generated. The phase velocity of the Lamb wave mode is related to the incident angle using the following equation [22]:

(2) $$\theta =\sin^{-1}\frac{c}{c_{p}}\;,$$

where θ is the angle at which the wave is generated or received, *c* is the speed of sound in the coupling medium (air in this study), and *c_p_* is the phase velocity of the generated Lamb wave mode in steel. Dispersion curves can be used to calculate the phase velocity related to a Lamb wave mode. The dispersion curve describes the relationship between wave velocity and frequency–thickness content for the solid medium [23]. Figure 3 presents the dispersion curve of the steel plate with the properties listed in Table 3.

The thickness of the steel plate is 4.8 mm, and the central frequency of the ultrasonic transducer is 0.4 MHz, which together result in the frequency–thickness (fd) value of 1.96 MHz-mm. As shown in Figure 3, only the fundamental S_0_ and A_0_ modes exist at this value. The phase velocities for the S_0_ and A_0_ fundamental modes are calculated as 4756 and 2640 m/s, respectively. By using the air velocity of 340 m/s and Equation (2), the angles required to generate the dominant S_0_ and A_0_ modes are calculated as 4° and 7°, respectively.

**Figure 4:**
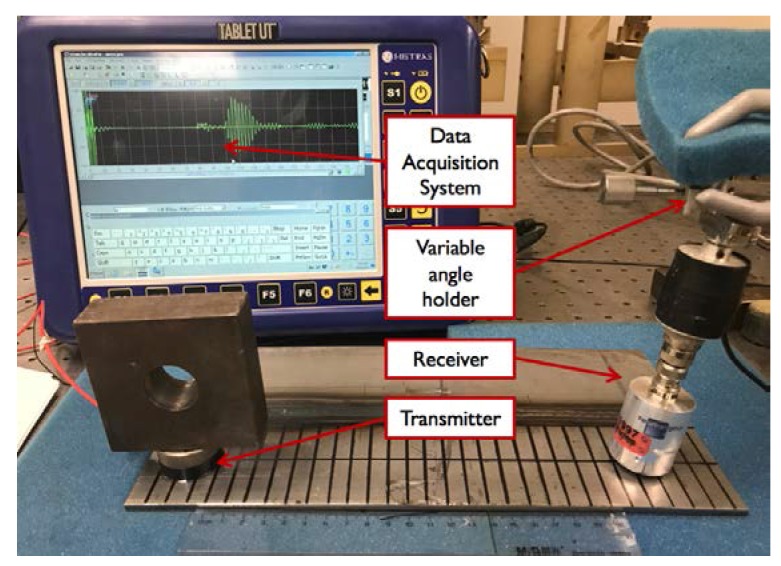
Experimental setup for the ideal angle identification.

A hybrid contact/noncontact measurement system is used to identify the ideal transducer angle experimentally. The experimental setup, as presented in Figure 4a, is composed of a Panametrics V101 (Olympus Scientific Solutions Americas Inc., Waltham, MA, USA) one inch diameter contact transmitter with a center frequency of 0.5 MHz, and an air-coupled noncontact receiver manufactured by Ultran Group (State College, PA, USA) with an active area diameter of 19 mm and center frequency of 0.4 MHz. The distance between the transducers is fixed at 210 mm to separate the A_0_ and S_0_ modes. In addition, a precise variable angle holder (Olympus Scientific Solutions Americas Inc., Waltham, MA, USA) is used at the receiver side to measure the angle.

**Figure 5:**
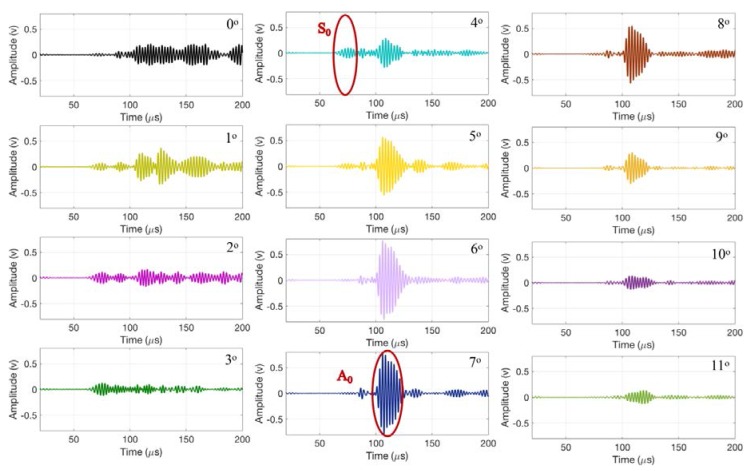
The recorded waveforms corresponding to receiver angles of 0° to 11°.

Figure 5 presents the time history signals corresponding to the receiver angles of 0° to 11°. The amplitude of the S_0_ mode is maximized at 4°. Similarly, the amplitude of the A_0_ mode is maximized at 7°. These results are in good agreement with the analytical values reported above.

### 2.3. Experimental Investigation of Weld Defects

**Figure 6:**
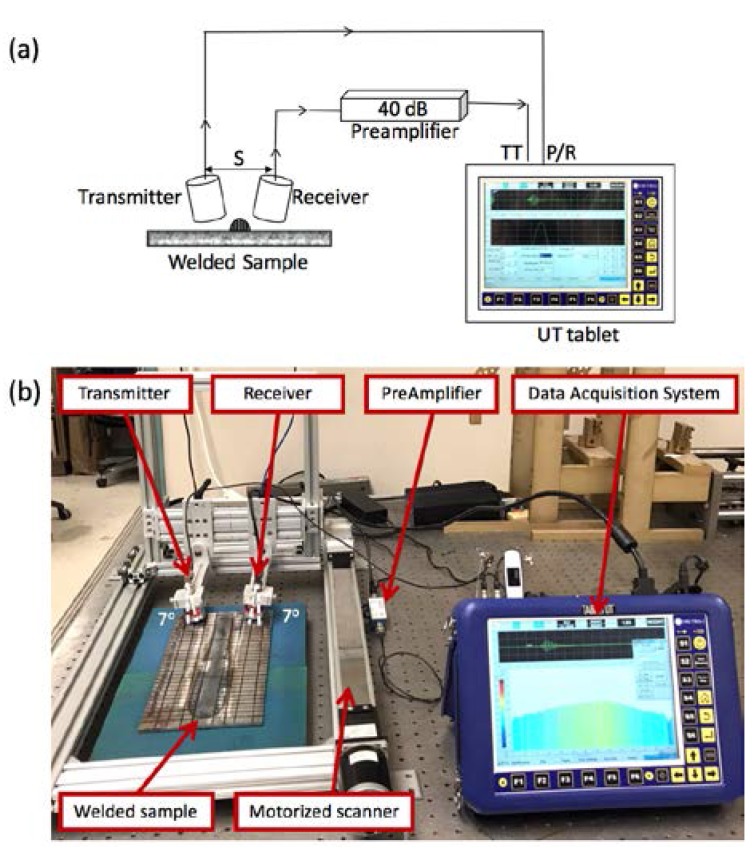
The experimental setup of air-coupled ultrasonic testing: (**a**) schematic; (**b**) photograph.

Figure 6 demonstrates the experimental setup used to investigate weld defects with the air-coupled ultrasonic method. The measurement system consists of two air-coupled ultrasonic transducers manufactured by Ultran Group with an active area diameter of 19 mm, center frequency of 0.4 MHz, and frequency bandwidth of ±0.117 MHz (down to −6 dB). The transducers are fixed at the required angle of 7° to get the dominant A_0_ Lamb wave mode as calculated in the previous section. As observed in Figure 5, a slight change in the transducer angle influences the ultrasonic signal. Therefore, the scanner is designed to keep the angles of the transmitting and receiving transducers consistent throughout the experiments. The distance (S) between the transducers is fixed at 60 mm to allow for a thorough inspection of the welded sample with minimum boundary reflections.

A portable dual-channel tablet UT manufactured by Mistras Group (Princeton Junction, NJ, USA) (with a sampling frequency of 100 MHz) was used to generate a two-cycle tone burst signal. First, the excitation signal was amplified with a gain of 52 dB (with a voltage amplitude of 400 volts), and then received through a preamplifier (designed by Mistras Group) with a gain of 40 dB to address the poor energy transfer of the air-coupled transducers as shown in Figure 6b, as the transmission loss from air to steel is approximately −45 dB [25]. To improve the signal to noise ratio, 200 waveforms were averaged and filtered with a passband of 0.2–10 MHz. 

**Figure 7:**
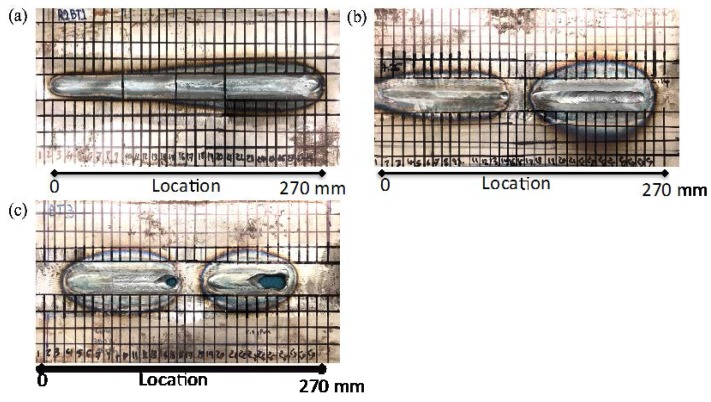
Weld coupons with different degrees of burn-through defect: (**a**) weld coupon No. 1; (**b**) weld coupon No. 2; and (**c**) weld coupon No. 3.

The steel plates were divided into 1 cm sections to undertake a more systematic inspection of the welded plates. Figure 7 shows welded samples with different degrees of burn-through. The transducers were attached to a motorized scanner developed by Industrial Measurement Systems (IMS, Aurora, IL, USA) as an autonomous monitoring device to allow for a B-scan through the length of the weld. The scanner moves over the plate, covering a length of 270 mm at a constant speed of 5 mm/s and collecting time domain waveforms every 2 mm, as shown in Figure 7. A Matlab (MATLAB 8.5, The MathWorks, Inc., Natick, MA, USA, 2015) script is used to extract various features from the time history data and their frequency spectra.

## 3. Results and Discussion

### 3.1. Identification of Weld Microstructure

**Figure 8:**
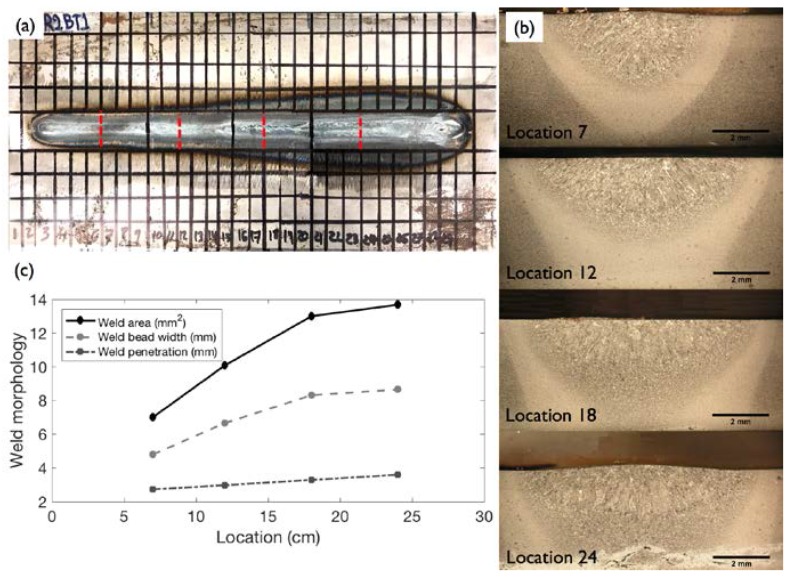
(**a**) Layout of weld coupon No. 1 showing the locations of the cross sections that were analyzed; (**b**) weld cross section characteristics and microstructures; and (**c**) weld width and weld area measurements.

Samples from weld coupon No. 1 were chosen for analysis of weld bead cross section changes and microstructure and correlation with the UT results. This coupon was selected because of the subtle changes in the weld microstructure compared to the noticeable differences observed in the other two coupons, as seen in Figure 7. The weld cross section samples were cut from four locations (see Figure 8) into small pieces of 2 × 2 × 0.48 cm^3^. The pieces were prepared using standard metallographic procedures and etched with a 2% Nital solution. The microstructures were examined using a stereomicroscope (Olympus Co., Tokyo, Japan). The weld bead width, penetration, and area were measured using ImageJ software (1.5i, National Institute of Health, Bethesda, MD, USA).

Figure 8 shows the cross section locations as well as the weld microstructure and the change in weld width, penetration, and area with welding current, respectively. The gradual increase in current resulted in increases in penetration, weld bead width, and overall weld area. Sample 4 of weld coupon No. 1 is defined as the onset of burn-through since there was initial melting on the backside of the plate, as shown in Figure 8b (Location 24). 

The heat input in Location 24/weld coupon No. 1 is 0.74 KJ/mm, which represents the onset of the burn-through defect, as compared to the other three locations (Locations 7, 12, and 18) where the weld bead remained within the steel plate cross section and the heat inputs were lower. The other weld coupons that were fabricated at larger heat inputs have increasing levels of burn-through-related damage, as seen particularly in Figure 7c. 

### 3.2. Correlation of UT Signals with Burn-Through Damage

**Figure 9:**
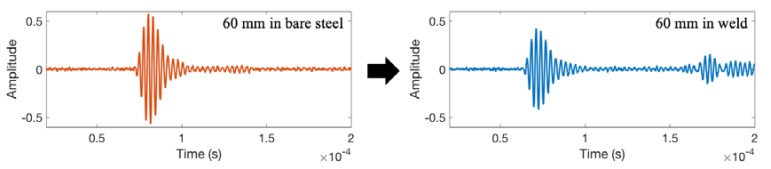
The comparison of the time domain waveforms for the signal with a travel distance of 60 mm in bare steel vs weld coupon No. 1.

To eliminate the initial excitation and multiple wave reflections, the signal window from 20 to 160 μs was considered. Figure 9 compares the shapes of the time domain waveforms for a signal travel distance of 60 mm in bare steel vs in welded steel. The wave arrival near 150 μs in the welded sample is through the boundary and not considered in the feature extraction. 

**Figure 10:**
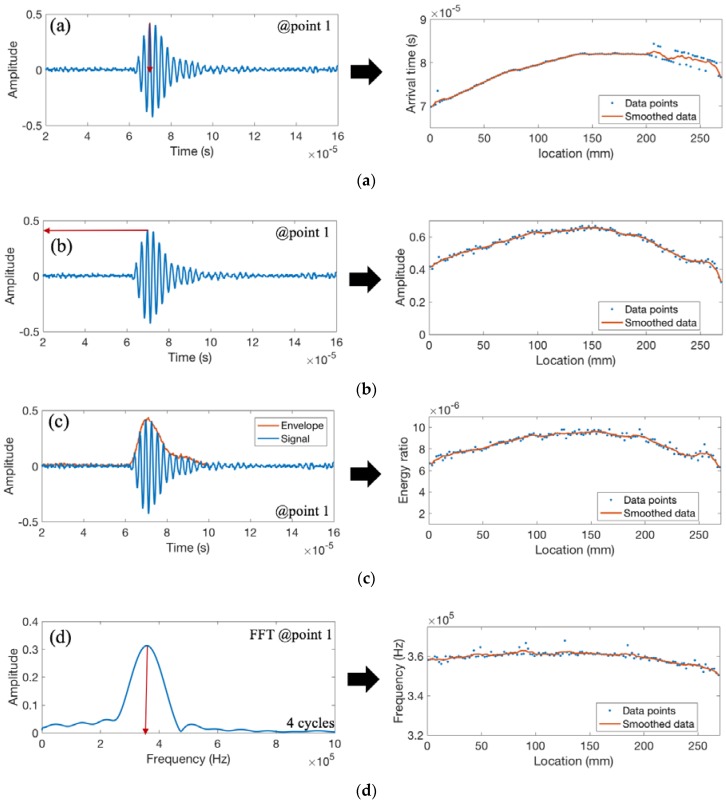
The recorded waveform and features extracted from weld coupon No. 1 corresponding to (**a**) the arrival time of the peak amplitude; (**b**) the peak amplitude; (**c**) the area under the envelope of the first arrived waveform; and (**d**) the peak frequency.

Figure 10 shows the recorded waveforms and the extracted features for weld coupon No. 1 with burn-through defect as shown in Figure 8b. Figure 10a shows the arrival time of the peak amplitude along the weld length. It is worth noting that the start and the end of the weld should be disregarded due to inconsistencies caused by the arc start and extinguishing of the arc at the end of the weld. The first part of the weld has insufficient penetration and the final part of the weld has excessive penetration. The sound weld is in the middle section (approximately 100–200 mm length). When there is insufficient penetration, the ultrasonic wave needs to go through two different materials—the base metal and the weld metal—which causes changes in arrival time due to the different properties and interfaces. When there is sound weld, the major part of the ultrasonic signal passes through the weld metal. The results show a sudden increase in the arrival time at the onset of the defect (at location 200 mm), which demonstrates a decrease in the velocity. This can be caused by irregularities in the wave path and/or partial Lamb mode conversions in the weld area. Figure 10b demonstrates the peak amplitude along the weld length, and Figure 10c shows the energy ratio feature calculated using the area under the envelope of the first arrived waveform. Both features (the energy ratio and peak amplitude) decrease with the increase of burn-through defect. The frequency shift feature (Figure 10d) is the frequency of the maximum amplitude calculated from the FFT(fast Fourier transform) of the first four cycles of the waveform. The frequency (decreasing as the weld width increases) is not the result of a shift in the wave propagation frequency but due to the attenuation effect [26] and/or partial wave conversion [19] at the interference of the base metal and the weld metal. As discussed in Section 2.1, a burn-through defect is caused by excessive penetration of weld to the base metal, which may cause holes through the base metal. For weld coupon No. 1, there is no open hole; however, excessive penetration is observed towards the end of the plate (see location 24 in Figure 8). Irregularities in the weld metal cause the scattering of ultrasonic waves, reducing the ultrasonic amplitude as well as causing inconsistent arrival time readings. In particular, the A_o_ mode is more sensitive to changes in the through thickness as it represents the flexural mode where the particle movement is perpendicular to the direction of wave propagation [27].

**Figure 11:**
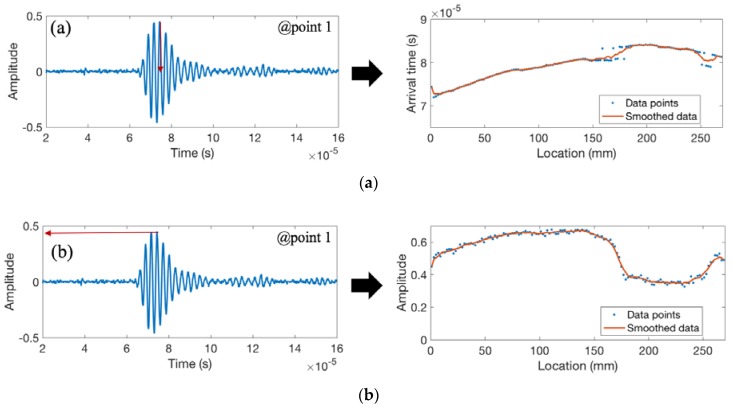

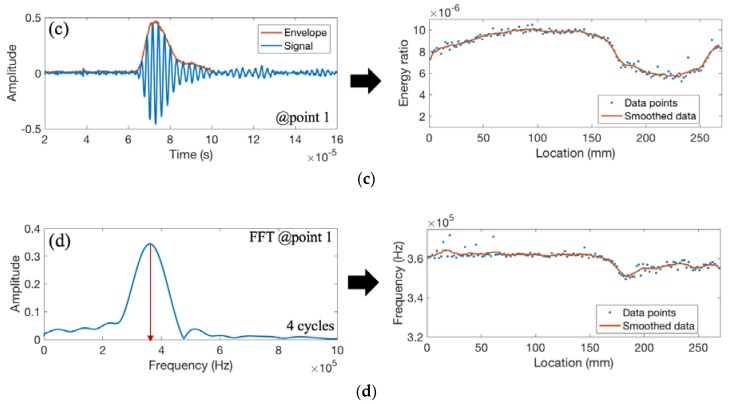
The recorded waveform and features extracted from weld coupon No. 2 corresponding to (**a**) the arrival time of the peak amplitude; (**b**) the peak amplitude; (**c**) the area under the envelope of the first arrived waveform; and (**d**) the peak frequency.

Figure 11 shows the recorded waveforms and the extracted features for weld coupon No. 2 with the burn-through defect. The defect happens in the second half of the plate (sample 2/coupon No. 2) which has a significant dip in the weld area (at location 190 mm). Similar to the previous example, the results show a sudden increase in the arrival time at the onset of the defect and a decrease in signal amplitude, energy, and frequency with the increase in the magnitude of the burn-through defect.

**Figure 12:**
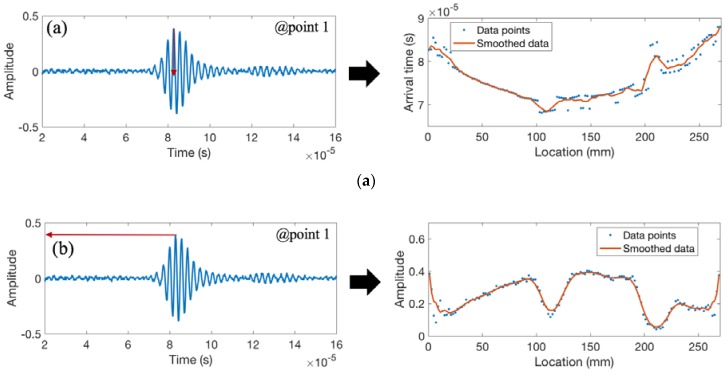

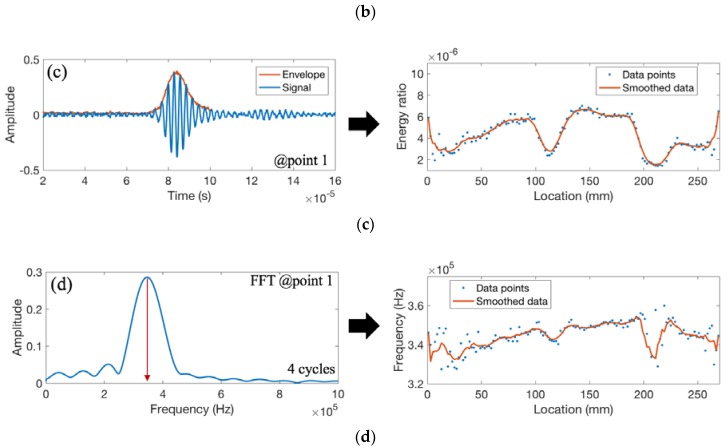
The recorded waveform and features extracted from weld coupon No. 3 corresponding to (**a**) the arrival time of the peak amplitude; (**b**) the peak amplitude; (**c**) the area under the envelope of the first arrived waveform; and (**d**) the peak frequency.

Figure 12 shows the recorded waveforms and the extracted features for weld coupon No. 3 with the burn-through defect. The burn-through defect in sample 2/coupon No. 3 has pierced a hole in the welded zone (at locations 110 and 210 mm). Similar to the previous samples, there is a sudden increase in the arrival time and a decrease in signal amplitude and energy with the increase in burn-through; however, due to the increase in the magnitude of the defect leading to heterogeneity in the cross section and inconsistency in the weld morphology, the signal strength is much lower.

**Figure 13:**
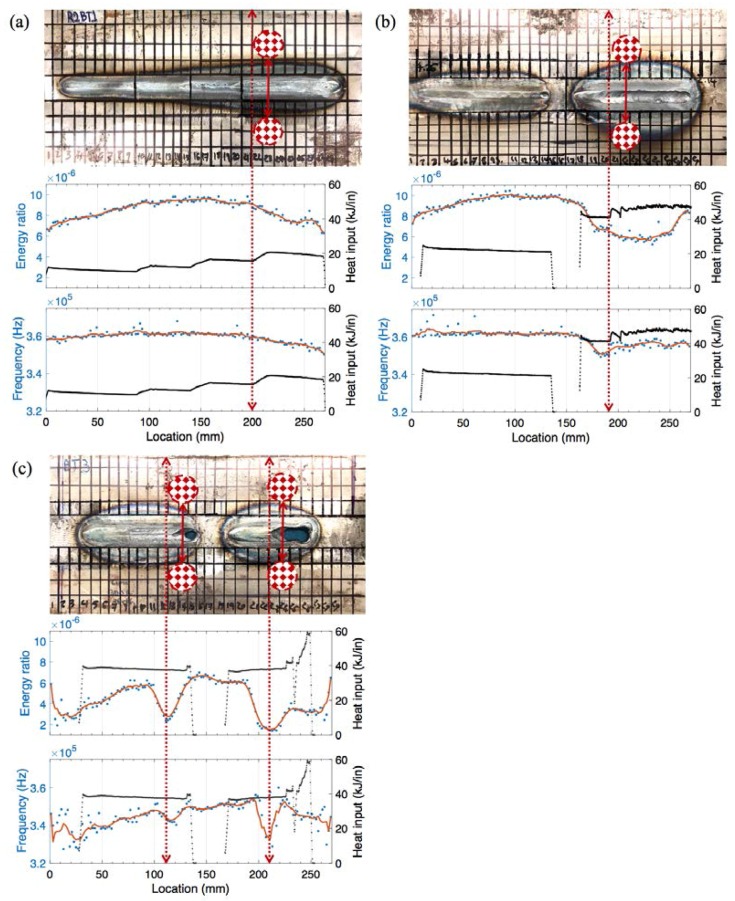
The correlation of UT features (energy ratio and peak frequency) and weld heat input with major changes in the weld microstructure corresponding to weld coupons (**a**) No. 1; (**b**) No. 2; and (**c**) No. 3, respectively.

Figure 13 shows the correlation of UT features (energy ratio and peak frequency) and weld heat input with significant changes in the weld microstructure corresponding to weld coupons Nos. 1 to 3. Energy ratio was selected to represent the time domain information as it includes both amplitude- and frequency-related characteristics within its calculation. For all the samples, the energy ratio and frequency values decrease with an increase in burn-through damage (see the dashed red lines on the plots). Energy ratio is more sensitive to weld size and penetration depth. For instance, the energy ratio increases with an increase in weld size and penetration, and then decreases with the presence of burn-through in sample No. 1 and No. 2 as observed at up to 150 mm of weld length (corresponding to the micrograph of location 18 in Figure 8). The frequency is only sensitive when burn-through damage is observed. The frequency value decreases below 360 kHz when burn-through damage occurs, which can be explained by the scattering of ultrasonic waves due to discontinuities in the microstructure and partial Lamb mode conversions. For sample No. 3, the energy ratio (<6 × 10^−6^) and frequency (<350 kHz) are the lowest due to the high current and complete burn-through damage throughout the weld. Two UT features can be used to identify burn-through damage and the welding parameters leading to burn-through damage. 

## 4. Conclusions

In this paper, a noncontact ultrasonic inspection method has been utilized to inspect welding burn-through defects that arise in gas tungsten arc welding. In this welding technique, excessive heat input and travel speed can cause the burn-through defect, leading to undesirable open holes in the welded plate. In this study, burn-through defects with different penetration levels were introduced by increasing the welding current or reducing the travel speed while keeping the other welding parameters constant. The Lamb-wave-based approach for airborne ultrasonic testing was then applied in order to correlate various ultrasonic features with significant changes in the weld microstructure. The results show that the burn-through defect leads to a larger volume of degraded weld zone, providing a resistance path for the wave to travel, which results in a lower velocity, energy ratio, and amplitude. Additionally, the wave energy disperses due to the increase of burn-through defect, resulting in higher attenuation. While the concurrent implementation of welding and ultrasonic testing raises other challenges such as the influence of magnetic field caused by the welding torch on ultrasonic waves in air, due to the nondestructive and noncontact nature of this technique, it is readily applicable to in-situ inspection of welding while the welded part is still in place. Two UT features—energy ratio and frequency—can be used to accept/reject the weld through detection of burn-through damage and identify the welding parameters causing the burn-through damage. While no filler metal is used in this study, it is expected that the relative changes in the UT features would be similar when filler metal is used if the weld morphology has similar characteristics (weld size, width, and heterogeneity through thickness). Future work arising from this study includes characterizing porosity and insufficient penetration in addition to burn-through, and developing machine learning algorithms to identify and distinguish the three major weld defects. 

## Figures and Tables

**Table 1:** Chemical composition of A36 [21].

ASTM Specification	Content %					
	C	Mn	P	S	Si	Cu
A36	0.25	–	0.03	0.03	0.40	0.20

**Table 2:** Different changes of Amperages and travel speed to obtain burn-through.

Weld Coupon	Sample	Current	Voltage	Gas Flow	Travel Speed	Tungsten Diameter	Heat Input	Visual Inspection
		(A)	(V)	(m^3^/h)	(mm/s)	(mm)	(kJ/mm)	
Onset burn-through due to variation of Amperages								
No. 1	1	160	8.41	0.99	3.39	3.175	0.40	Good Weld
	2	200	8.41	0.99	3.39	3.175	0.50	Intermediate heat input
	3	250	8.41	0.99	3.39	3.175	0.62	High heat input
	4	300	8.41	0.99	3.39	3.175	0.74	Initiation of burn-through
Burn-through due to variation of travel speed with normal Amperage								
No. 2	1	160	9.65	1.02	1.80	3.175	0.86	Burn-through with small amount of melting material
	2	160	9.99	1.02	0.91	3.175	1.76	Burn-through with large amount of melting material
Burn-through due to variation of travel speed with high Amperage								
No. 3	1	300	12.32	1.02	2.54	3.175	1.46	Burn-through with small hole
	2	300	11.84	1.02	2.33	3.175	1.53	Burn-through with large hole

**Table 3:** The material constants of structural steel [24].

Property	Values			
Density (kg/m^3^)	7850			
Young’s modulus (MPa)	200 × $10^{3}$			
Poisson’s ratio	0.33			
Lame constants (MPa)	λ = 150 × $10^{3}$		μ = 75 × $10^{3}$	
Murnaghan constants (MPa)	l = −300 × $10^{3}$	m = −620 × $10^{3}$		n = −720 × $10^{3}$
